# The Validity and Reliability of a New Simple Instrument for the Measurement of First Ray Mobility

**DOI:** 10.3390/s20082207

**Published:** 2020-04-14

**Authors:** Pedro V. Munuera-Martínez, Priscila Távara-Vidalón, Manuel A. Monge-Vera, Antonia Sáez-Díaz, Guillermo Lafuente-Sotillos

**Affiliations:** 1 Departamento de Podología, University of Seville, s/n. 41009 Seville, Spain; priscilatavara16@gmail.com (P.T.-V.); glafuente@us.es (G.L.-S.); 2 Department of Design Engineering, University of Seville, 741011 Seville, Spain; amonge@us.es; 3 Department of Statistics, Axioma Comunicaciones, 41016 Seville, Spain; asaez@axiomacomunicaciones.com

**Keywords:** first ray, mobility, foot, measurement, quantification, device

## Abstract

Several methods have been described to quantify the first ray mobility. They all have certain disadvantages (great size, sophistication, or lack of validation). The objective of this work was to study the validity and reliability of a new instrument for the measurement of first ray mobility. Anterior-posterior radiographs were obtained from 25 normal feet and 24 hallux valgus feet, with the first ray in a neutral position, maximally dorsiflexed and maximally plantarflexed. The first ray mobility was radiographicaly measured in both groups, and was also manually examined with the new device. A cluster analysis determined whether normal and hallux valgus feet were correctly classified, and a graphic analysis of Bland-Altman was performed to compare the radiographic and manual measurement techniques. Based on the radiographs, the first ray mobility only showed significant differences in dorsiflexion between both groups (*P* = 0.015). First ray dorsiflexion, plantarflexion and total range of motion measured with the new device were different between both groups (*P* = 0.040, *P* = 0.011 and *P* = 0.006, respectively). The silhouette measure of the cohesion and separation coefficients from the cluster analysis was greater than 0.50 for the dorsiflexion, plantarflexion and total range of motion obtained from the radiographs and from the new device. The Bland-Altman graph suggested that 96% of the data presented agreement between both measurement methods. These results suggested that the new instrument was valid and reliable.

## 1. Introduction

The normal motion of the first ray has received the attention of many authors over the years and has been related to different foot pathologies [1,2,3,4]. Different methods have previously been used to quantify this range of motion (ROM). Morton [5,6] described the first manual maneuver to examine the mobility of the first ray. This consisted of applying a dorsal force under the first metatarsal head with one hand, whereas the other hand held the lesser metatarsal heads. Root et al. [7] slightly varied this technique. With the hands positioned as described by Morton, they suggested moving the first metatarsal head toward a maximally dorsiflexed position, and toward a maximally plantarflexed position, and then compare both movements. These authors proposed that, clinically, the normal ROM of the first ray should be 5 mm of dorsiflexion and 5 mm of plantarflexion, with hypermobility identified when dorsiflexion was greater than plantarflexion.

Obviously, simplicity is an important requisite for any clinical test. Unfortunately, manual examination of the first ray ROM lacks sufficient diagnostic accuracy [8], and may be subject to great inter-rater variability. Therefore, instruments to quantify the mobility of the first ray in a valid and reliable way are needed. In the 1990s, Klaue et al. [9] and Glasoe et al. [10] developed valid instruments to measure dorsiflexion of the first ray. Klaue’s instrument consists of a frame attached to an ankle-foot orthosis and from the frame suspended a caliper so that it rested on the first metatarsal to record motion as the examiner dorsiflexes the first ray maximally. Glasoe’s device consists of an ankle-foot orthosis that stabilizes the hindfoot, being the patient sat with their ankle and forefoot immobile in a neutral position, while a separate platform hold the first metatarsal level with the lesser metatarsals to mark the initial neutral position. This device produces a force displacement measurement, and records movement from a probe placed on the head of the first metatarsal. In spite of these instruments having been used in various studies, [11,12,13,14,15,16] they have some inconveniences such as, for example, being too complex, being large, not quantifying plantarflexion (which would be useful to compare with dorsiflexion), or not reflecting the combined movements of dorsiflexion-inversion and plantarflexion-eversion [4,17]. These are possible reasons why they are not used in daily clinical practice [8]. More simple instruments have been subsequently created aiming at solving some of the aforementioned inconveniences [18,19]. However, some studies have reported only limited concordance and data reliability [16,20].

**Figure 1:**
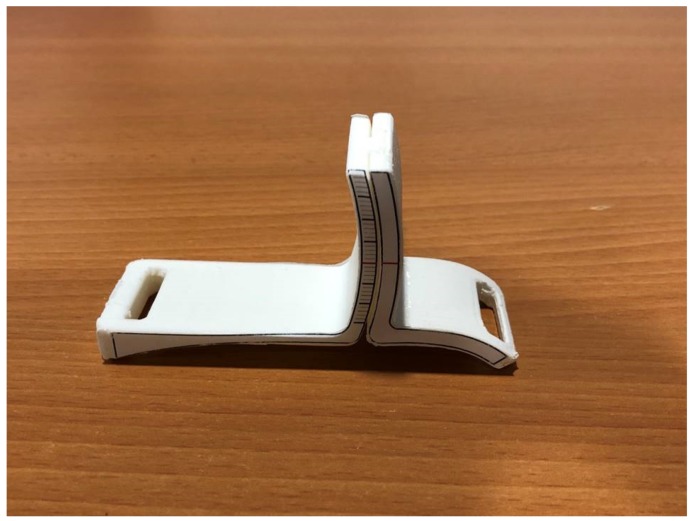
The new instrument for the measurement of the first ray mobility.

In a recent study a mathematical formula describing a ‘normal curve’ of the first ray static ROM has been reported [21]. This formula was used to design a new instrument to measure the dorsiflexion and plantarflexion of the first ray (patented in Spain with patent number ES201500721, manufactured and distributed by Fresco Podología S.L., Barcelona, Spain). It consists of a small, light, simple plastic instrument, easy to use in daily practice, and which reflects the combined movements of the first ray (Figure 1). The main objective of the present study was to prove the validity and reliability (intra- and inter-rater) of this new instrument. Secondly, the triplanar first ray ROM was compared between feet with normal first rays and feet with hallux abducto-valgus.

## 2. Materials and Methods

### 2.1. Participants

Forty nine volunteers (40 women) were included in the study. They were adult volunteers or patients with an average age of 36.75 ± 21.34 years old attending the Podiatry Clinical Area of the University of Seville. The participants were recruited between November 2018 and June 2019.

Informed consent was received from all the participants and the rights of the subjects were protected. Authorization was obtained from the Podiatry Clinical Area of the University of Seville, and from the Ethical Research Committee of the University Hospitals “Virgen Macarena” and “Virgen del Rocío” of Seville, Spain.

Participants who served as the control group were healthy volunteers or patients who presented for elimination of hyperkeratosis or treatment of nail disorders. The inclusion criteria for the control group were: adults with normal feet according to the criteria for normalcy established by Kirby [22] (feet that function normally during gait, have no history of significant trauma or surgery, and have no pain or significant deformity), normal first ray (morphologically and functionally), and with first ray normal mobility. For the hallux valgus (HV) group these were: adults with hallux valgus whose first ray had to be visually classified as hypermobile via manual examination. People who had experienced injuries affecting the first ray mobility, surgical intervention in the first ray, or had suffered inflammatory, metabolic processes, degenerative or neuromuscular diseases affecting their feet, were excluded from the study. When two different examiners could not clearly identify the points to mark in some radiographs, those participants were also excluded. Only one foot per person was included. As the way an individual foot functions is partly dependent on the whole person, the degree of association between both feet (right and left) could be greater than the degree of association between different subjects [23]. When the HV was bilateral, the most severe one was chosen. When either both feet presented the same grade of severity of HV, or presented normal first ray mobility bilaterally, the choice was made by flipping a coin in the presence of two researchers. 

To include a participant in the study, a manual examination of the mobility of the first ray was carried out via the classic clinical maneuver described by Root et al. [7]. Two examiners had to agree separately in their valuation of the first ray as ‘normal’ using this maneuver, or as ‘hallux valgus’ using the Manchester scale [24,25]. ‘Normal mobility’ of the first ray was recorded when dorsiflexion and plantarflexion were present and approximately equal. 

### 2.2. Sample Size Calculation

The variable representing the total range of motion of the first ray in the sagittal plane, obtained from pilot data of the first six participants (mean ± SD = 8.29 ± 2.37), was used as a reference to calculate the minimum sample size employing the following formula to compare mean values between two groups (control and HV):

(1) $$n=\frac{{2s}^{2}{\left(z\frac{\alpha }{2}+z\beta \right)}^{2}}{d^{2}}$$

where *s* is the standard deviation, *α* is the type I error (0.05), *β* is the type II error (0.16), and *d* is the minimum difference to be detected (it was selected 2 because the difference of dorsiflexion between normal and HV first ray was hoped to be 2 mm). Therefore, the final equation is:

(2) $$=\frac{{2s}^{2}{\left(z\frac{\alpha }{2}+z\beta \right)}^{2}}{d^{2}}=\frac{{2\cdot 2.37}^{2}{\left(1.96+0.84\right)}^{2}}{2^{2}}=22.0108≅22$$

Thus, at least 22 people were needed in each group. Finally, 25 participants constituted the control group and 24 the HV group.

### 2.3. Study Design 

**Figure 2:**
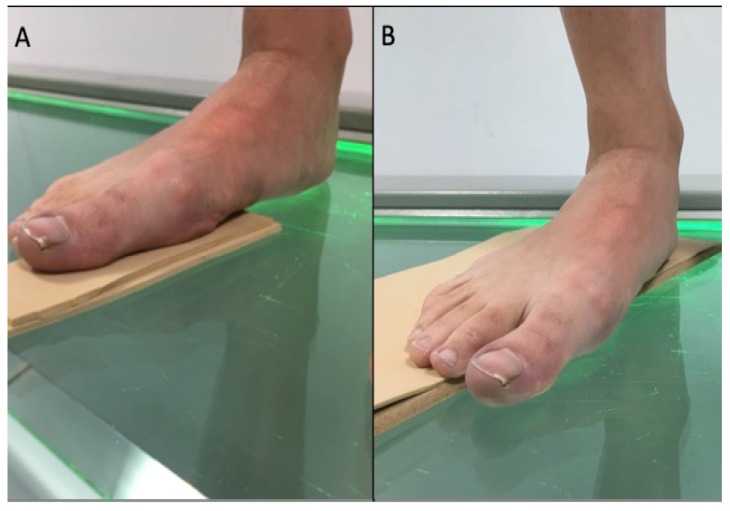
Foot positioning for AP radiographs (**A**): First ray dorsiflexion; (**B**): First ray plantarflexion.

This was a clinical measurement, cross-sectional study. When a participant was included in the study, radiographs were done using the modified Coleman’s test described by Fritz and Prieskorn to quantify the first ray maximum dorsiflexion and plantarflexion [26]. Previously, blocks (2 to 5 mm thick) were progressively placed under the first metatarsal or the lesser metatarsals to quantify the maximum dorsiflexion and plantarflexion, respectively (Figure 2), with the participant in a standing position [21]. 

Three anterior-posterior (A-P) radiographs of the foot were obtained, with the first ray maximally dorsiflexed, maximally plantarflexed and in a neutral position, using the aforementioned blocks. These radiographs were used to quantify the range of motion of the first ray in the sagittal, frontal and transverse planes. 

### 2.4. Measurements

**Figure 3:**
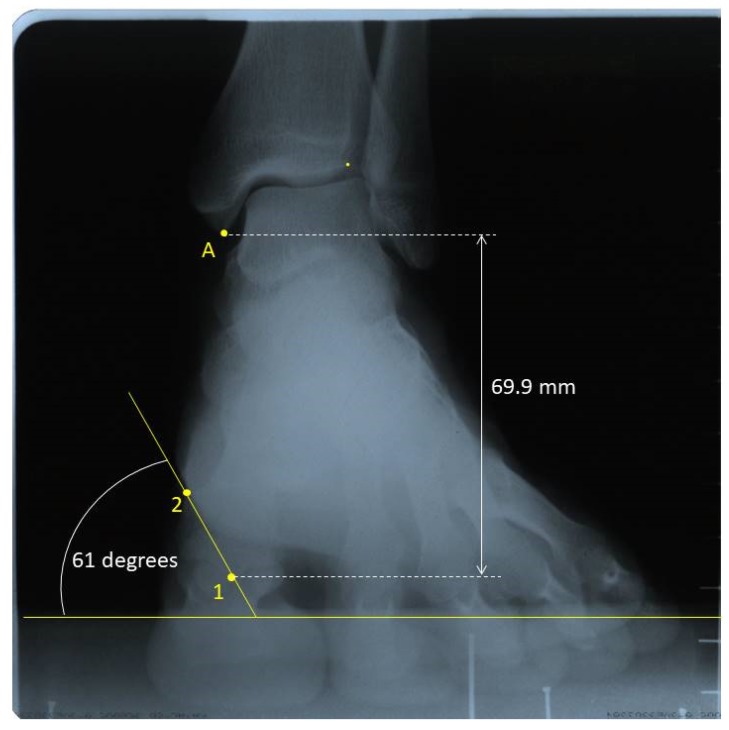
Radiographic measurements in one foot with first ray in neutral position. Point 1: intersesamoid crest; Point 2: superomedial tubercle of the first metatarsal head. Point A: the most distal tip of the tibial malleolus.

Radiographs were digitalized using an Epson Expression 1680 Pro^®^ scanner (Seiko Epson Corporation, Tokyo, Japan) that can explore images in positive films. For radiographic measurements, the AutoCAD^®^ software was used (AutoCAD 2016; Autodesk Inc, San Rafael, CA, USA). One point in the intersesamoid crest (point 1) and another one in the superomedial tubercle of the first metatarsal head, where the medial collateral ligament of the first metatarsophalangeal joint begins (point 2), were marked. A third point was drawn in the distal tip of the medial malleolus (point A), which was used as a fixed point (Figure 3).

To record the first ray mobility in the sagittal plane the distance from point A to point 1 was measured in millimeters in the three positions. The difference of this distance between the neutral and the dorsiflexed position was recorded as the dorsal displacement of the first ray, and the difference of this distance between the neutral and the plantarflexed position was recorded as the plantar displacement of the first ray.

To measure the movement in the frontal plane in the three positions, an angle formed between the following two lines were drawn: one line between points 1 and 2, and the other being a horizontal line parallel to the ground. When this angle increased, the mobility was recorded as inversion, and vice versa. To obtain the first ray displacement in the transverse plane, the distance from point 1 to a vertical line crossing point A was measured in millimeters, also in the three positions. When point 1 was in the medial side of this vertical line, that is, closer to the second metatarsal, positive signed values were registered. When this point fell on the lateral side of the vertical line, that is, farther from the second metatarsal, negative signed values were registered.

**Figure 4:**
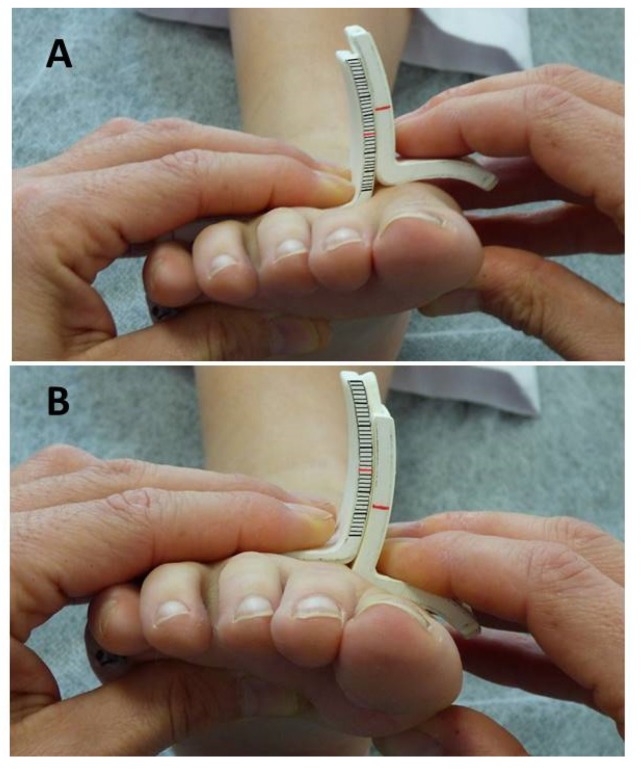
Measurement of first ray dorsiflexion (**A**) and plantarflexion (**B**) with the new instrument.

In order to validate the new device, data obtained from the radiographs and those obtained manually where used in the subsequent statistical analysis. To evaluate the mobility of the first ray with the new instrument, the participants lay supine on the examination table with their ankle in a relaxed position and the subtalar joint in a neutral position. The examiner clamped with one hand the instrument’s long arm and the head of the lesser metatarsals and with the other hand the instrument’s short arm and the head of the first metatarsal. The head of the first metatarsal was then moved to maximal dorsiflexion and the displacement was recorded in millimeters. The same procedure was repeated to register the maximal plantarflexion (Figure 4).

In order to determine the intra-rater and inter-rater reliability, this measurement protocol was carried out twice on all the participants by two different researchers. Each measurement by the same examiner was separated by a period of 10 to 30 days. Prior to commencing the study, the two raters met during two separate 1-hour sessions to discuss and practice techniques to ensure concordance.

### 2.5. Data Analysis

The statistical analysis was carried out via the SPSS Statistics® software for Windows^®^, version 22 (IBM, Corp, Armonk, NY, USA). The displacement of the first ray in the three planes, obtained through the x-ray images, was described and compared between the normal and HV feet. The Shapiro-Wilk test was used to determine if data followed a normal distribution, and the Mann-Whitney U test or the Student’s T test for independent samples was employed to carry out the comparisons. All the values of *P* < 0.05 were considered statistically significant.

Intra-rater and inter-rater reliability for first ray mobility recorded with the new instrument was evaluated using intraclass correlation coefficients (two-way mixed, average measures, consistency). ICC 95% confidence intervals were added to determine its dispersion.

In order to validate the new device, a two-phase cluster analysis was carried out to determine whether the belonging of a subject to a group (normal or HV feet) could be defined. The result was considered good when the silhouette measure of cohesion and separation coefficient was greater than 0.5. In addition, a Bland-Altman graphic analysis was performed to compare the two measurement techniques (manual and radiographic). The limits of tolerance were calculated through the 95% confidence interval; that is, the measurements were considered to be concordant if at least 95% of them were located within these limits of tolerance.

Finally, the ROC curve method was applied to analyze the predictive capacity of the new instrument measurements to correctly identify patients with or without HV. Values of the area under the curve (AUC), sensitivity, specificity, positive predictive value (PPV), and negative predictive value (NPV) were determined.

## 3. Results

### 3.1. Participants’ Characteristics

**Figure 5:**
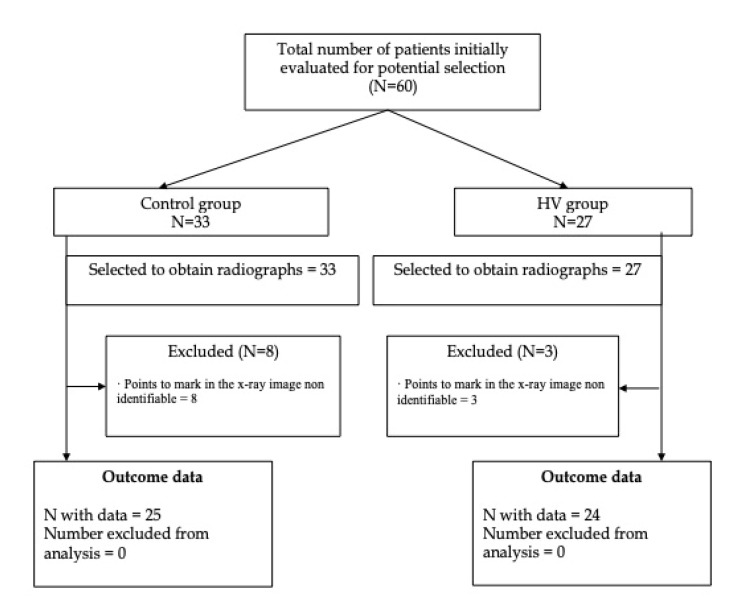
Flow diagram.

Although 60 volunteers initially took part, 11 participants were excluded because two different examiners could not clearly identify in the radiographs the points to mark (three with HV and eight with a normal first ray). Finally, 49 participants were included (25 in the control group and 24 in the HV group), with an average age of 36.75 ± 21.34 years old (range 19–79) and a BMI of 23.62 ± 4.66 kg/m^2^ (Figure 5). Twenty-eight right feet and 21 left feet were included.

### 3.2. Group Differences

The control group was made up of 7 men and 18 women, mean age 22.28 ± 3.81 years, BMI 22.11 ± 3.91. The HV group was made up of two men and 22 women, mean age 51.83 ± 21.69 years, BMI 25.19 ± 4.92. The groups were similar in sex distribution (*P* = 0.078) and side (*P* = 0.451), however, the age and body mass were different (*P* < 0.001 and *P* = 0.017, respectively).

The radiographic data of the first ray mobility in the sagittal, frontal and transverse planes, in both groups are shown in Table 1. The only movement showing statistically significant difference between the control and the HV group was dorsiflexion. The neutral position of the first ray, that is, the position of the first metatarsal head with the participant standing in a relaxed position in order to take the x-ray image, showed some differences between groups (Table 2). In the sagittal plane initial position, a higher value implies a less dorsiflexed position (more distance between the tibial malleolus and the intersesamoid crest), and vice versa. The first ray in HV group presented a more dorsiflexed position than in normal feet, although the difference did not become significant at a 95% level of significance (*P* = 0.060). In the frontal plane initial position, a higher value means a more inverted position (a higher angle between the line connecting points 1 and 2, and a horizontal line), and vice versa. The first metatarsal head in HV group presented a significantly more everted position than in normal feet (*P* = 0.005). In the transverse plane initial position, a positive value means that the point 1 was located within the tibial side of the vertical line crossing point A, and vice versa. Both groups showed a similar initial position in the transverse plane (*P* = 0.795).

For comparisons of the first ray mobility measured with the new device, only data obtained by the more experienced examiner were used. The mean first ray dorsiflexion with the new device was 6.49 ± 0.97 mm for the control group, and 7.20 ± 1.37 mm for the HV group. The mean first ray plantarflexion was 5.26 ± 0.89 mm for the control group, and 5.99 ± 1.04 mm for the HV group. The total range of motion was, therefore, 11.75 ± 1.48 mm for the control group, and 13.20 ± 2.00 mm for the HAV group. Significant differences were observed between groups (dorsiflexion: *P* = 0.040; plantarflexion: *P* = 0.011; total ROM: *P* = 0.006).

### 3.3. Reliability

The intrarater reliability of manual measurement of the first ray mobility with the new device is shown in Table 3. Good to excellent reliability across the two raters was found for dorsiflexion and plantarflexion in both the control and the HV groups.

The inter-rater reliability of manual measurement of the first ray mobility is shown in Table 4. Excellent reliability across the two raters, the less experienced and the most experienced, was found for dorsiflexion and plantarflexion in both the control and the HAV groups.

### 3.4. Validity

**Figure 6:**
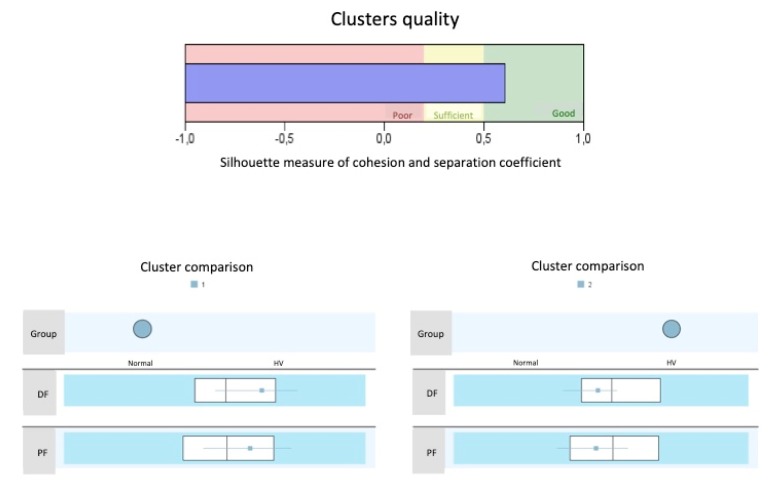
Cluster graphic. A silhouette measure of cohesion and separation coefficient of 1 means that all cases are in their cluster centers. A value of -1 means that all cases are in the centers of other clusters to which they do not belong. A value of 0 means that, on average, the cases are equidistant between the center of their own conglomerate and the center of another nearby conglomerate. Therefore, a result in the “Good” zone means that the data strongly evidence the structure of the conglomerates. A result in the “Sufficient” area means that the data show this cluster structure in a less evident way, and a result in the “Poor” area reflects that the data does not provide significant evidence of the cluster structure. DF = Dorsiflexion; PF = Plantarflexion.

The silhouette measure of cohesion and separation coefficient was 0.61 for the values of dorsiflexion and plantarflexion separately obtained from the radiographs, and 0.58 for the values from the new device. When the total ROM was considered, the silhouette measure of cohesion and separation coefficient was 0.69 for the data obtained from the radiographs and 0.72 for the values from the new device (Figure 6). In both cases this means a good classification. As shown in Table 5, when the total ROM of first ray is less than 11.6 millimeters, the first ray could be classified as normal. In contrast, with 12.8 mm or more, the feet could be classified as HV. Therefore, there is a small interval of uncertainty (1.2 mm) in which cases normal and HV first rays could be confused.

**Figure 7:**
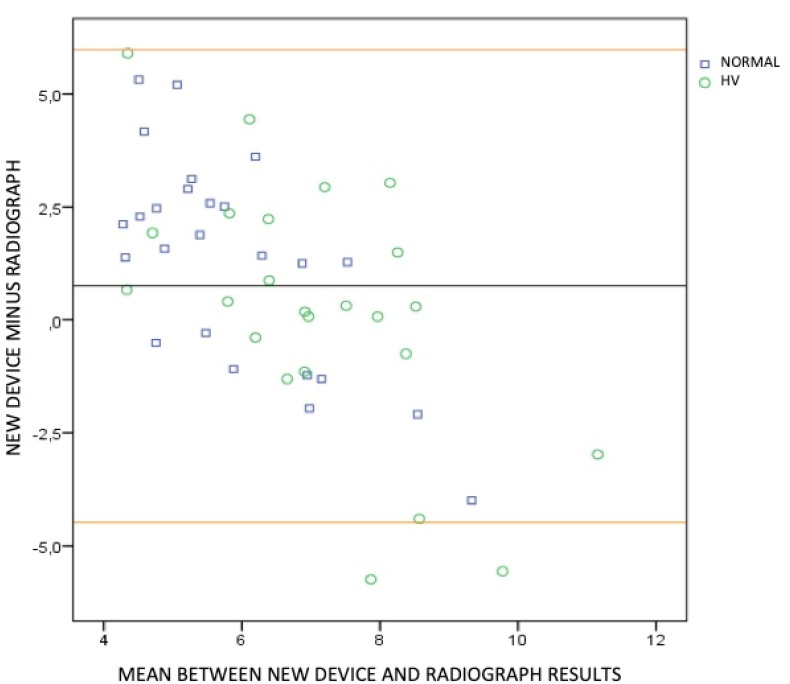
Bland-Altman graph. The dorsiflexion measured with the new device minus the dorsiflexion measured with radiographs was calculated for each participant. The mean and the standard deviation of the obteined results were then calculated (0.75 ± 2.67). This mean and standard deviation were used to calculate the tolerance limits (Lower limit: 0.75 − 1.96 × 2.67 = –4.48. Upper limit: 0.75 + 1.96 × 2.67 = 5.98). These limits are represented by the upper and lower horizontal lines in the graph. The midline represents the mean of the differences obtained between the dorsiflexion measured with the new device and the dorsiflexion measured with radiographs (0.75).

In order to know the agreement between the different measurements (X-ray and device), the Bland-Altman graph was obtained using the variable dorsiflexion (Figure 7). It shows that 96% of the cases were within the tolerance limits, that is, 96% of the data presented agreement between both measurement methods. 

The values of the area under the curve which showed the best sensitivity and specificity for the three measurements (dorsiflexion, plantarflexion and total ROM) are given in Table 6, together with the positive and negative predictive values. In addition, the most optimal values considered to be the cut-off point at which one participant would belong to control or HV group, are shown. ROC graphics may be consulted as Supplementary Material.

## 4. Discussion

One of the objectives of this work was to compare the mobility of the first ray between feet with and without HV. As far as the authors know, this is the first study that quantifies the ROM of the first ray in the sagittal, frontal and transverse planes via radiograph images in subjects with and without HV. According to the results obtained, the total sagittal displacement of the first ray was not significantly different between normal and HV feet. However, dorsiflexion was. This means that the sagittal ROM was similar, but the distribution of the dorsal and plantar amount of motion was different, as the normal feet showed less dorsiflexion, and more (though not significantly different) plantarflexion than the HV feet. Previous researchers also found greater dorsal displacement of the first ray in feet with HV than in normal feet [4,9,19,27,28,29,30]. Otherwise, mobility in the frontal and transverse planes did not show significant differences between both types of feet. 

The 3-dimensional mobility of the first ray has been previously studied in patients with HV [4,17,27,31], reporting mobility in the sagittal and frontal planes and, to a lesser extent, in the transverse plane (adduction). In the present study adduction was only observed in HV feet when the first ray dorsiflexed. However, these data showed a high variability (high standard deviations and wide 95% CIs). Although there was not a significant difference between the normal and the HV feet, it was observed that the HV feet presented more motion in the transverse plane than the normal feet, maybe as a result of the instability related to this deformity. Further research is needed to quantify the displacement of the first ray in the transverse plane during dorsiflexion and plantarflexion.

The combination of motion observed in the sagittal and frontal planes was in concordance with what is most widespread and accepted; that is, dorsiflexion with inversion and plantarflexion with eversion, both in normal and HV feet. Hicks [32] noted that the first ray made dorsiflexion-inversion and plantarflexion-eversion, and Ebisui [33] sustained that the first ray made dorsiflexion and inversion movements during the pronation of the foot, and plantarflexion and eversion movements during supination. Also Sarrafian [34] and Root et al. [35] supported this theory. Using modern medical image technology, other recent studies also reported dorsiflexion and inversion taking place together [4,17]. The new instrument used to manually measure the first ray mobility reflects these movements, thanks to the curvature of the vertical arms.

On the other hand, there are authors who uphold that during subtalar pronation, the dorsiflexion of the first ray is accompanied by eversion [36,37,38,39], although these studies include participants with HV, not with normal first rays. Ota et al. [40] reported significant eversion in HV patients compared to control group patients, by using computed tomography-based 3D analysis to investigate the first metatarsal torsion in HV patients. In the present study, a ‘more everted’ position of the first metatarsal head has been observed in the HV participants (*P* = 0.005). This could be the result of an osseous functional adaptation of the first ray to the valgus position of the hallux in the HV deformity. This condition should not affect the new device records, as it is not influenced by the initial position of the first metatarsal head, but by the movement from the neutral position. 

Kelso et al. [41] examined the first ray ROM in 24 specimens and they also observed the combined mobility of dorsiflexion-inversion and plantarflexio-eversion. Motion in the transverse plane was insignificant. In the sagittal plane, the mean ROM was 12.38 ± 3.4 mm (range 2.72−17.86). In the frontal plane, the mean ROM was 8.23 ± 4.12 degrees (range 3.3–19.37). They determined that each millimeter of movement in the sagittal plane was accompanied by 0.77 degrees of frontal plane rotation. Yet, other studies reported different data [42]. By dividing the degrees of rotation in the frontal plane obtained in the present study (5.84) between the millimeters of displacement in the sagittal plane (8.51), the result is that for each millimeter of displacement in the sagittal plane the first ray made a rotation of 0.69 degrees in the frontal plane. These results are similar to Kelso et al’s. data [41] 

Another objective of this study was to determine the validity and reliability of a new measure instrument to quantify the dorsal and plantar mobility of the first ray. According to the results of this study the new instrument has proved to be valid and reliable. 

In the last decades many methods have been proposed to measure first ray mobility. However, they are not used for daily clinical practice. The reasons for this are discussed below. Two of the most referenced in the literature due to the high validity and reliability reported are the devices described by Klaue et al. [9] in 1994, validated in 2005 [13], and that designed by Glasoe et al. [43] in 1998, validated in 2000 [15]. However, other instruments have been proposed. Rogers and Cavanagh [44], created an instrument that measured the vertical displacement of the first ray from beneath the first metatarsal head by mechanically delivering an external load. Wallace and Kilmartin [18] designed a handheld ruler to measure the mobility of the first ray. As far as the authors know, these devices have not been validated. Lee and Young [19] described a plastic ruler to be placed on the dorsal surface of the first and second metatarsal heads while delivering a manual force to dorsiflex or plantarflex the first ray. Glasoe et al. [16] compared data registered with Lee’s ruler and data collected with Glasoe’s device. They found the ruler measures to be unreliable, whereas the measures made with Glasoe’s device were highly reliable. In addition, they observed no agreement between the device and ruler measurements of dorsal displacement of the first ray. Lastly, Whitney [45,46] and Greisberg et al. [47] designed rulers to be used on the plantar surface of the forefoot, but these were not validated. In addition, it has been reported that measures acquired on the plantar surface could introduce error due to compression of the fat pad [43].

The results of the cluster analysis for the values of dorsiflexion and plantarflexion separately obtained from the radiographs, as well as when the total ROM was considered, and those for the values from the manual examination, suggest that the new device provides a good classification of the first ray (normal or HV). The Bland-Altman graph also showed high agreement between the different measurements (X-ray and device), showing that 96% of the data presented agreement between both measurement methods.

Although it was not an objective of this study to explore the diagnostic capacity of the new instrument, it was considered to be informative to report sensitivity, specificity and predictive values for the movements measured with this device. All predictive values obtained, except one (PPV for dorsiflexion) were 60% or higher, which imply good capacity to correctly classify patients. According to these results, a subject showing more than 6.75 mm of dorsiflexion and more than 5.08 of plantarflexion, or more than 12.25 mm of total ROM, could be classified as belonging to the HV group. It is needed to state that not every cases of HV curse with hypermobility of the first ray. Participants of this study were selected if they visually presented an increase of first ray mobility using the traditional manual maneuver of examination, but not always this hypermobility is present. Further research would be needed to establish the normal mobility of first ray in wider samples, and also in some of the most frequent foot pathologies.

This new instrument has characteristics that may solve some of the aforementioned disadvantages related to previously described instruments. Firstly, its simplicity, lightness, size and ease of use, make it possible to be used in daily clinical practice. Moreover, it reflects the combined movement of the first ray in the sagittal and frontal planes. A mathematical formula describing a ‘normal curve’ of the first ray static ROM has been recently reported [21]. This formula was used to design the new instrument to measure the dorsal and plantar mobility of the first ray. Obviously, patients may have different mobility values of the first ray in each plane, and it would be too complicated to use a customized measure instrument. It is necessary to establish a base to continue building research and knowledge of it, regarding the quantification of the mobility of the first ray. Measuring the mobility of the first ray could potentially help clinicians quantify the thickness of certain elements for foot orthoses, choose among different corrective surgical techniques, or compare results before and after surgery, for example.

This study has certain limitations. For example, the anatomic orientation of the first TMTJ was not taken intro account, as two-dimensional images have been used to value 3-dimensional elements. The potential risk of errors was decreased by following a rigorous X-ray protocol. Previous investigations demonstrated that when the same protocol is employed to obtain X-ray images, the differences with reality are not significant, at least with respect to the first metatarsal-digital segment [48]. Another limitation is that the normal ROM of the first ray was quantified using the first metatarsal head mobility; it was not differentiated according to the joints of the medial foot column. However, the methodology used is relevant for the manual evaluation of the first ray in the clinical daily activity. 

The method used in this study to quantify the first ray mobility in the transverse plane through x-ray images could have not been the most appropriate. The vertical line used as a reference (crossing the most distal tip of the tibial malleolus) could vary its position in the radiograph with foot pronation due to the lower limb internal rotation that occurs together with subtalar pronation [49,50,51]. Further research is necessary to quantify the mobility of the first ray in the transverse plane when it dorsiflexes and plantarflexes. 

Finally, it must be taken into account that only normal first rays and HV have been included, so the results may vary with other conditions (i.e., hallux rigidus or plantarflexed first ray). 

## 5. Conclusions

A new device for the measurement of first ray mobility has been presented. It has characteristics such as simplicity, lightness, its small size and ease of use that facilitate its use in daily clinical practice. The results of the statistical analysis suggest that it showed a good validity, as it correctly classified 96% of the participants, and provided good concordance with radiographic measures. In addition, it showed good to excellent intra- and inter-rater reliability. 

Regarding the first ray mobility in both groups, dorsiflexion was accompanied by inversion and plantarflexion was accompanied by eversion. The first ray in the HV feet showed a total ROM similar to that of the normal feet, but dorsiflexion was significantly greater. The first metatarsal head showed a more everted neutral position in the HV feet than in the normal feet, which could mean that an osseous functional adaptation of the first metatarsal head to the valgus position of the hallux in the HV deformity had occurred. 

## Figures and Tables

**Table 1:** Radiographic data of the first ray range of motion in the sagittal, frontal and transverse planes in both groups, and significance (*P*-value).

Variable	Control Group (Mean ± SD)	HV Group (Mean ± SD)	P-Value	Difference 95% CI
Dorsiflexion (mm)	5.19 ± 2.32	7.03 ± 2.76	0.015	−3.30–0.38
Plantarflexion (mm)	3.32 ± 2.27	2.80 ± 1.84	0.383	−0.67−1.71
Total sagittal movement (mm)	8.51 ± 3.41	9.83 ± 2.81	0.147	−3.12−0.48
Inversion (degrees)	2.68 ± 4.09	2.71 ± 3.22	0.979	−2.15–2.09
Eversion (degrees)	3.16 ± 2.91	2.12 ± 3.98	0.303	−0.96–3.03
Total frontal movement (degrees)	5.84 ± 5.20	4.83 ± 3.78	0.444	−1.62–3.63
Movement in the transverse plane during dorsalflexion * (mm)	−0.64 ± 5.86	1.69 ± 6.01	0.656	−3.10–4.87
Movement in the transverse plane during plantarflexion * (mm)	−4.11 ± 6.06	–5.00 ± 7.74	0.176	−5.74–1.08
Total transverse movement (mm) †	4.11 ± 6.06	6.69 ± 9.01	0.244	−1.82–6.98

* A negative value means that point 1 moved away from the second metatarsal (abduction). † These are absolute values, from the initial position to the mean abducted position in normal feet, and from the mean adducted position to the mean abducted position in HV feet.

**Table 2:** Initial position of the first metatarsal head. The values of the sagittal plane position are millimeters from tibial malleolus (point A) to point 1 (the intersesamoid crest). A higher value implies a less dorsiflexed position (more distance between the tibial malleolus and the intersesamoid crest), and vice versa. The values of the frontal plane are degrees between a horizontal line and a line joining points 1 (the intersesamoid crest) and 2 (the superomedial tubercle). A higher value means a more inverted position (a higher angle between the line connecting points 1 and 2, and a horizontal line), and vice versa. The values of the transverse plane are millimeters from point 1 to a vertical line crossing point A (a positive value means that the point 1 was located within the tibial side of the vertical line crossing point A, and vice versa).

Variable	Control Group Mean ± SD (95% CI)	HV GroupMean ± SD (95% CI)	P-Value
Sagittal plane	77.42 ± 8.19 (74.04–80.80)	72.69 ± 8.99 (68.90–76.49)	0.060
Frontal plane	53.80 ± 5.67 (51.45–56.15)	50.00 ± 4.74 (48.00–52.00)	0.005
Transverse plane	10.64 ± 6.48 (8.10–13.18)	9.55 ± 5.38 (7.40–11.70)	0.525

**Table 3:** Intrarater reliability (ICC) from day 1and 2 (separated by a minimum of 10 and a maximum of 30 days) for each first ray movement measured with the new device.

	ICC (95% CI)	
	Rater 1	Rater 2
Control group		
Dorsiflexion	0.883 (0.794–0.942)	0.892 (0.810–946)
Plantarflexion	0.907 (0.837–0.954)	0.895 (0.816–0.948)
HV group		
Dorsiflexion	0.872 (0.691–0.963)	0.808 (0.537–0.945)
Plantarflexion	0.917 (0.799–0.976)	0.792 (0.597–0.940)

**Table 4:** Inter-rater reliability (ICC) from raters 1and 2 in day 1 for each first ray movement measured with the new device.

INTER-RATER			
ICC		DF	PF
Rater1-Rater2	Normal	0.928 (0.878–0.963)	0.940 (0.898–0.969)
	HV	0.919 (0.818–0.976)	0.925 (0.832–0.978)
	Total	0.932 (0.893–0.961)	0.932 (0.893–0.961)

**Table 5:** Values of the total ROM of the first ray used in assembling the conglomerates for predicting normal or HV feet.

Group	Total First Ray ROM (mm)
Normal feet	10.5 to 11.6
Interval of uncertainty	11.6 to 12.8
HV feet	12.8 to 14.6

**Table 6:** Area under curve (AUC), sensitivity, specificity, positive predictive value (PPV) and negative predictive value (NPV) of the new device measurements. DF: Dorsiflexion; PF: Plantarflexion; ROM: Range of Motion.

	DF	PF	TOTAL ROM
**AUC (95% CI)**	0.656 (0.501–0.811)	0.721 (0.577–0.865)	0.704 (0.558–0.851)
**Cut-off point (mm)**	6.75	5.08	12.25
**Sensitivity (%)**	66.7	75.0	70.8
**Specificity (%)**	56.0	52.0	60.0
**PPV (%)**	59.3	60.0	63.0
**NPV (%)**	63.6	68.4	68.2

## Supplementary Materials

Supplementary materials can be accessed at https://www.mdpi.com/1424-8220/20/8/2207/s1.
