# Supplementary figures and images for: The Validity and Reliability of a New Simple Instrument for the Measurement of First Ray Mobility
Source: Sensors (Basel). 2020 Apr 14;20(8):2207. doi: 10.3390/s20082207 (PMC7218899; doi:10.3390/s20082207)

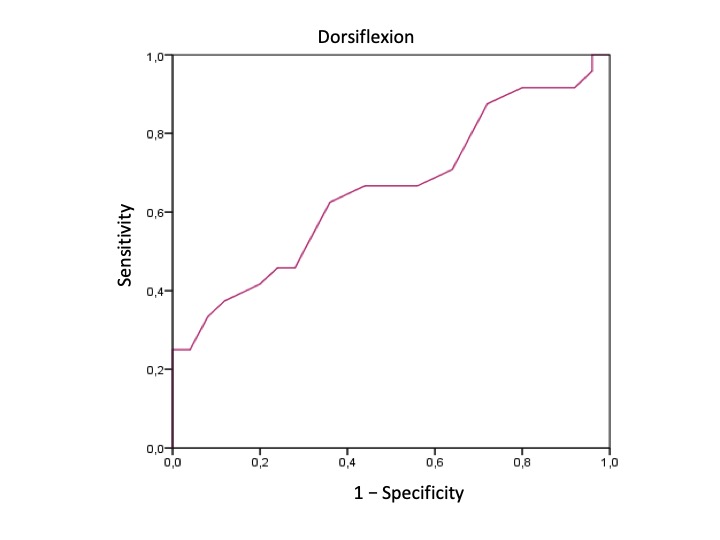

Supplement: Supplementary file 1 [file sensors-20-02207-s001.zip › ROC curve for Dorsiflexion.jpg]

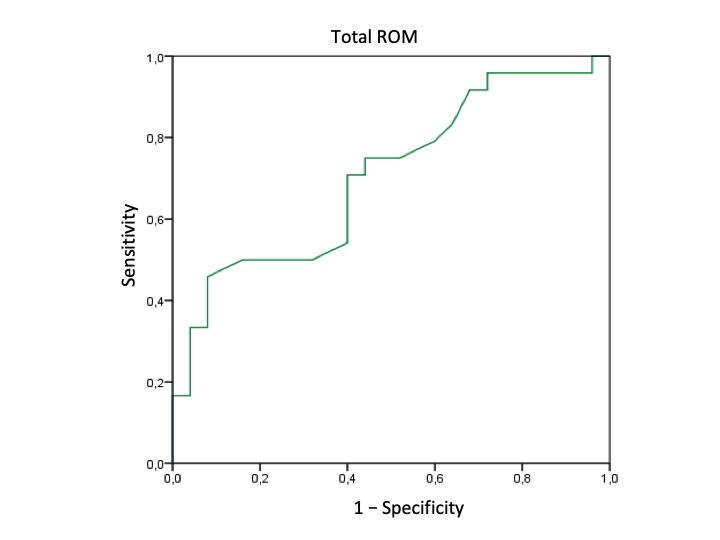

Supplement: Supplementary file 1 [file sensors-20-02207-s001.zip › ROC curve for Total ROM.jpg]

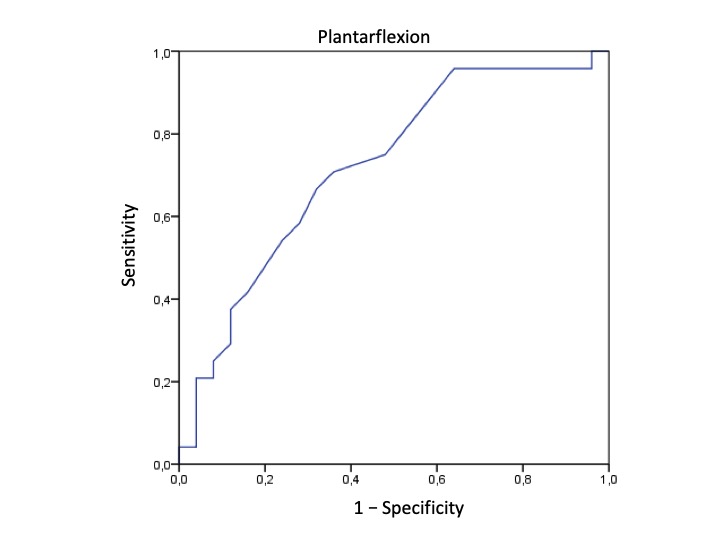

Supplement: Supplementary file 1 [file sensors-20-02207-s001.zip › ROC curve for Plantarflexion.jpg]
